# Characterization of 14-3-3-ζ Interactions with Integrin Tails

**DOI:** 10.1016/j.jmb.2013.05.024

**Published:** 2013-09-09

**Authors:** Roman Bonet, Ioannis Vakonakis, Iain D. Campbell

**Affiliations:** Department of Biochemistry, University of Oxford, South Parks Road, Oxford OX1 3QU, United Kingdom

**Keywords:** wt, wild type, HSQC, heteronuclear single quantum coherence, NMR, nuclear magnetic resonance, ITC, isothermal titration calorimetry, integrin cytoplasmic domains, protein–protein interactions, X-ray crystallography, NMR, ITC

## Abstract

Integrins are a family of heterodimeric (α+β) adhesion receptors that play key roles in many cellular processes. Integrins are unusual in that their functions can be modulated from both outside and inside the cell. Inside-out signaling is mediated by binding adaptor proteins to the flexible cytoplasmic tails of the α- and β-integrin subunits. Talin is one well-known intracellular activator, but various other adaptors bind to integrin tails, including 14-3-3-ζ, a member of the 14-3-3 family of dimeric proteins that have a preference for binding phosphorylated sequence motifs. Phosphorylation of a threonine in the β2 integrin tail has been shown to modulate β2/14-3-3-ζ interactions, and recently, the α4 integrin tail was reported to bind to 14-3-3-ζ and associate with paxillin in a ternary complex that is regulated by serine phosphorylation.

Here, we use a range of biophysical techniques to characterize interactions between 14-3-3-ζ and the cytoplasmic tails of α4, β1, β2 and β3 integrins. The X-ray structure of the 14-3-3-ζ/α4 complex indicates a canonical binding mode for the α4 phospho-peptide, but unexpected features are also observed: residues outside the consensus 14-3-3-ζ binding motif are shown to be essential for an efficient interaction; in contrast, a short β2 phospho-peptide is sufficient for high-affinity binding to 14-3-3-ζ. In addition, we report novel 14-3-3-ζ/integrin tail interactions that are independent of phosphorylation. Of the integrin tails studied, the strongest interaction with 14-3-3-ζ is observed for the β1A variant. In summary, new insights about 14-3-3-ζ/integrin tail interactions that have implications for the role of these molecular associations in cells are described.

## Highlights

• Integrin tails are important for bidirectional signaling across the membrane.
• 14-3-3-ζ binding to integrin tails has been studied using biophysical techniques.
• Residues outside the 14-3-3 binding motif contribute to affinity in α4 but not in β2.
• Phosphorylation-independent 14-3-3-ζ interactions with integrin tails are reported.

## Graphical abstract

**Figure:**
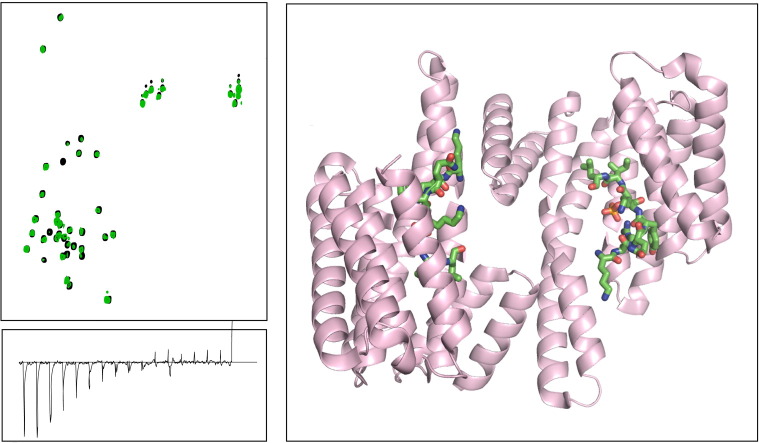

## Introduction

Integrins are membrane-spanning heterodimeric receptors, associated with a wide-range of normal functions, including cell adhesion, migration and differentiation, as well as disease.^1,2^ They are formed by α- and β-subunits composed of large extracellular (ecto) domains, a transmembrane domain and a usually short (13–70 residues) C-terminal cytoplasmic domain.^3^ The C-terminal domains are flexible “tails” that act as hubs for numerous protein–protein interactions^4–6^ that control various inside-to-outside and outside-to-inside signals. To date, knowledge of interacting partners and regulatory mechanisms is more advanced for β-integrin than for α-integrin tails. Characterized β-integrin tail binding proteins include talin,^7,8^ kindlin,^9,10^ filamin^11^ and 14-3-3.^12^

The 14-3-3 family consists of highly conserved acidic proteins of ~ 30 kDa molecular size that are expressed in all eukaryotic cells. In humans, seven distinct 14-3-3 isoforms (β, γ, ε, η, σ, τ and ζ) have been described, all of which form homodimers or heterodimers. They are involved in the regulation of many signaling pathways, including cell cycle progression, programmed cell death and cytoskeletal dynamics. The family is also associated with a number of human diseases, such as cancer and neurological disorders.^13,14^ 14-3-3 proteins were initially described as phosphor-serine/threonine binding modules with two consensus recognition motifs, classified as binding “mode-1” (RSXpSXP) and “mode-2” (RXF/YXpSXP).^15^ Subsequently, divergent binding modes have been described including affinity to unphosphorylated motifs.^16,17^ X-ray structures of numerous 14-3-3 isoforms and complexes have revealed considerable detail about the various binding modes.^18,19^

Recently, the structure of 14-3-3-ζ in complex with a phosphorylated peptide from the β2 integrin tail was determined.^20^ Another study linked 14-3-3-β with β1 and β3 integrins, but no structural details were provided.^21^ An association between 14-3-3-ζ and α4 was recently reported by Deakin *et al.*,^22^ and evidence was presented for a ternary complex with 14-3-3-ζ/α4 and paxillin, a focal adhesion protein that serves as a platform for the binding of numerous proteins.^23^ In addition, previous studies suggested that an association between paxillin and the α4 integrin tail is essential for regulating cell migration.^24–26^ It was proposed that this interaction is stabilized by 14-3-3-ζ in a ternary complex that accelerates cell migration.^22^ The binding of 14-3-3-ζ to α4 was also observed to depend on phosphorylation of α4 serine 1011.^22,27^

Here, we aimed to gain insight into the association of integrin cytoplasmic tails with 14-3-3 proteins. Our initial focus was characterizing the interaction between the α4 integrin tail and 14-3-3-ζ. We first solved the crystal structure of 14-3-3-ζ in complex with a phosphorylated α4-derived peptide and defined its binding mode. The use of complementary techniques, particularly NMR (*n*uclear *m*agnetic *r*esonance) and ITC (*i*sothermal *t*itration *c*alorimetry), revealed that the 14-3-3-ζ/α4 interaction is dependent not only on phosphorylation but also on residues outside the central 14-3-3-ζ binding motif. We found no evidence for a ternary 14-3-3-ζ/paxillin/α4 complex or a binary paxillin/α4 complex. We also carried out a biophysical characterization of β-integrin tail interactions with 14-3-3-ζ. All β-integrin tails tested bound to 14-3-3-ζ in a phosphorylation-independent manner through an epitope proximal to the transmembrane domain. In the case of β2 integrin tail, this region is well separated from the previously characterized phosphorylation-dependent 14-3-3-ζ binding site. Studies of 14-3-3-ζ variants provided further clues about the key determinants of the interactions.

## Results

### 14-3-3-ζ binding to the α4 integrin cytoplasmic tail

**Fig. 1:**
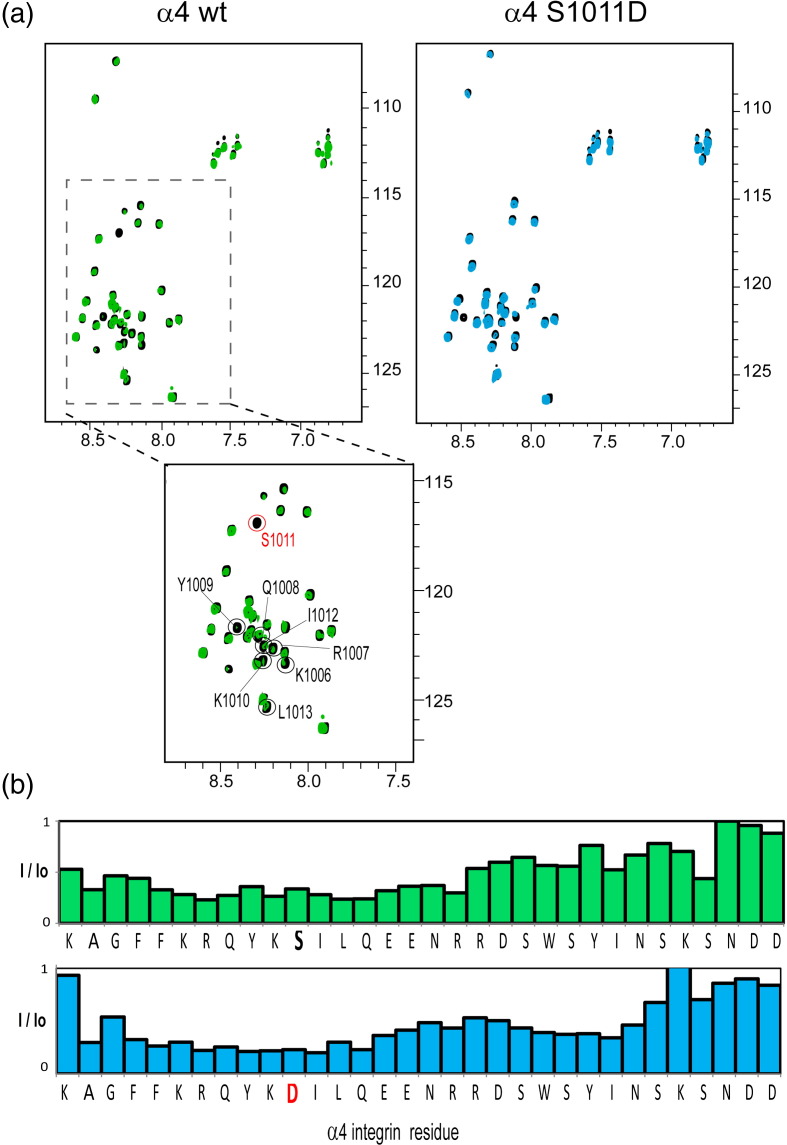
NMR studies of the α4/14-3-3-ζ interaction. (a) ^15^N–^1^H HSQC spectra of ^15^N-labeled 0.1 mM α4 wt (left) and S1011D (right) in black and after the addition of unlabeled 0.1 mM 14-3-3-ζ (overlaid in green and blue, respectively). In a close-up of the α4 wt spectrum below, resonances corresponding to the residues observed in the crystal structure are indicated in circles and the phosphorylatable serine is highlighted in red. (b) The intensity ratio (*I*/*I*o) in the presence (*I*) or absence (*I*o) of 14-3-3-ζ of α4 resonances shown in (a) for the wt (top) and S1011D variant (bottom). The intensity ratios were calculated using the CCPN Analysis software. The substituted serine residue is highlighted in red.

We used the α4 phospho-mimetic, S1011D, to explore the phosphorylation dependence of 14-3-3-ζ binding to the consensus RQYKSIL motif in the α4 tail.^22^ NMR-monitored titrations of ^15^N-labeled full-length α4 integrin tails, wild type (wt) or S1011D, with unlabeled 14-3-3-ζ produced changes of α4 resonances. As shown in Fig. 1a, addition of 14-3-3-ζ causes a significant decrease in α4 amide resonance intensities, indicative of an interaction. The extent of resonance intensity decrease is high across the whole α4 sequence but is more pronounced in the N-terminal region where the 14-3-3-ζ binding motif is located (Fig. 1b). Although the characteristics of the NMR spectra (severe broadening on addition of 14-3-3-ζ) make it difficult to extract precise information about binding affinities, they clearly show that a specific interaction between 14-3-3-ζ and α4 exists even in the absence of phosphorylation.

**Fig. 2:**
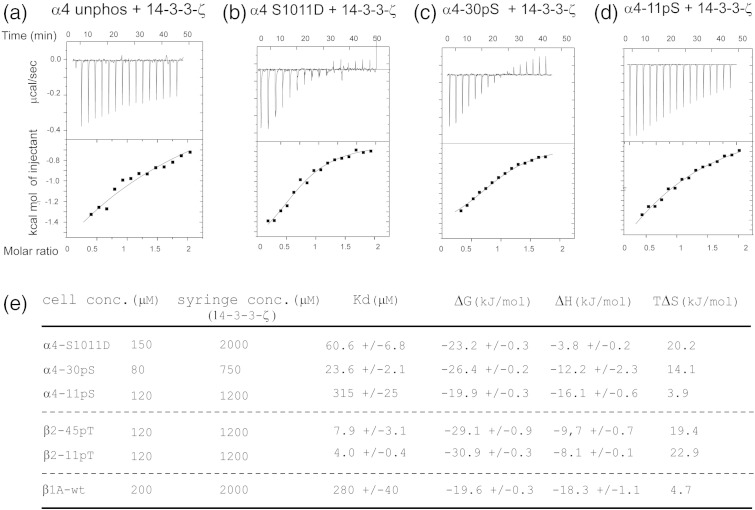
ITC studies of the α4/14-3-3-ζ interaction. (a) ITC sensorgram of in-cell 120 μM α4 wt sample injected with 1.2 mM 14-3-3-ζ; the interaction is weak, in the millimolar range, and an accurate fit could not be obtained. (b) ITC sensorgram of in-cell 150 μM α4 S1011D sample injected with 2.0 mM 14-3-3-ζ; thermodynamic parameters from the fit are shown in (e). (c) ITC sensorgram of in-cell 80 μM α4-30pS phospho-peptide injected with 0.75 mM 14-3-3-ζ. (d) ITC sensorgram of in-cell 120 μM α4-11pS phospho-peptide injected with 1.2 mM 14-3-3-ζ. (e) ITC affinity values and thermodynamic parameters of integrin tail fragments for the 14-3-3-ζ interaction.

The affinities of 14-3-3-ζ/α4 interactions were measured by ITC for the α4 wt and S1011D samples, as well as for two synthetic peptides containing phospho-serine residues α4-30pS and α4-11pS (see Table 1 for peptide nomenclature). Typical data are shown in Fig. 2a–d, and the resulting affinity values are summarized in Fig. 2e. The α4 wt binding to 14-3-3-ζ is relatively weak; thus, a *K*_d_ could not be determined by ITC. However, the α4 S1011D construct had a *K*_d_ value of approximately 60 μM, and the affinity of the phospho-serine containing α4-30pS was also strong (*K*_d_ ~ 24 μM). These results are consistent with a prominent role for phosphorylation in the regulation of 14-3-3 interactions and show that the phospho-serine-to-aspartic substitution is a suitable phospho-mimetic for the 14-3-3-ζ/α4 association. Crucially, we observed that α4 residues outside the consensus 14-3-3 binding motif are also necessary for efficient association, as illustrated by the 14-3-3-ζ/α4-11pS interaction that gave a *K*_d_ value of over 300 μM (an ~ 15-fold reduction in affinity compared to α4-30pS).

### Structure determination of the 14-3-3-ζ/phospho-α4 integrin tail complex

**Fig. 3:**
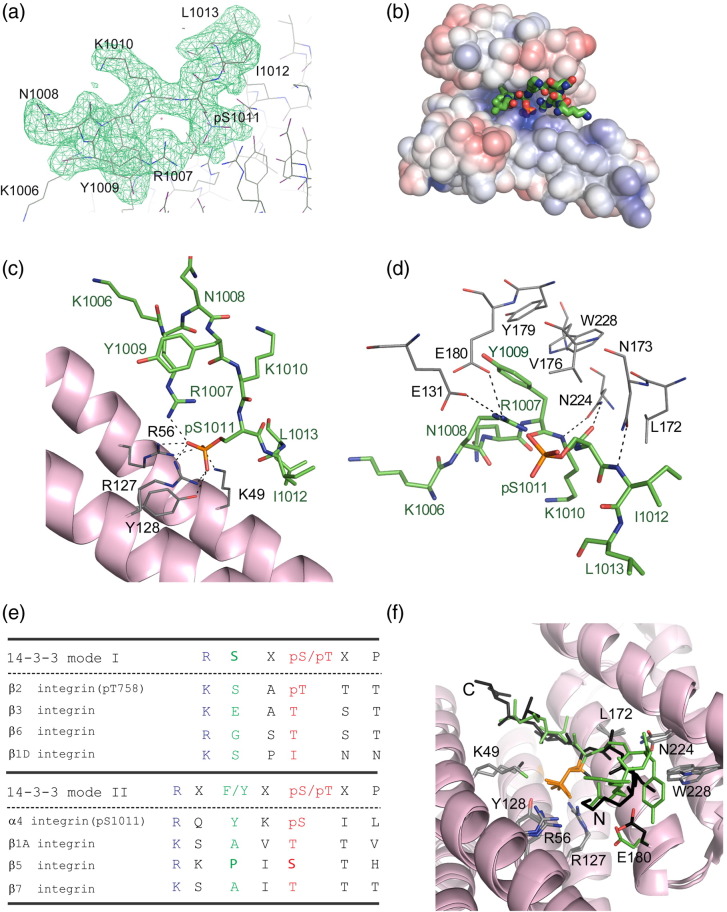
Details of the 14-3-3-ζ/α4 phospho-peptide interaction and comparison of the 14-3-3-ζ/α4 and 14-3-3-ζ/β2 binding modes. (a) Electron density omit map (2*F*_o_ − *F*_c_) of the α4 peptide region at σ = 2. The map was calculated using simulated annealing refinement in PHENIX^28^ after excluding the peptide and nearby atoms of 14-3-3-ζ. Peptide residues are labeled. (b) Representation of the electrostatic potential on the solvent-accessible surface of a 14-3-3-ζ monomer showing the basic pocket where the peptide phosphate group is accommodated. The α4 peptide is shown in green ball-and-stick representation, with the phosphate group in orange. The electrostatic surface was generated by the APBS software^29^ as a plug-in in PyMOL with chosen values of electrostatic potential of ± 5 kT/e for the representation. (c) Representation of the contacts between the phosphate group from the α4 peptide and the conserved basic pocket K49-R56-R127. The two additional hydrogen bonds with Y128 and R1007 from the peptide are also shown. The main chain of 14-3-3-ζ is displayed in light pink, the residues that bind the peptide phosphate group are displayed in gray and the α4 peptide chain is displayed in green. (d) Representation of the main 14-3-3-ζ residues (in gray) other than those in the basic pocket that make contacts with the peptide. Hydrogen bonds are represented by broken lines. Peptide residues are also represented, in green. (e) Alignment of integrin tail sequences showing the regions with reported (α4 and β2)^20,22^ or putative (other integrin tails) 14-3-3 mode-I and mode-II binding motifs. (f) Superimposition of the α4 (in green) and β2 (in black) integrin peptides from the structure presented here and Protein Data Bank 2V7D,^20^ respectively; the phosphate groups are indicated in orange. Some side chains of 14-3-3 residues important for the binding are also displayed in gray, except for E180, which has different orientations in the two complexes and is colored to match the corresponding integrin peptide.

The ITC observation that α4-30pS has a relatively high affinity led to the successful crystallization and structure determination of a complex with 14-3-3-ζ. Crystals were obtained in space group *C*222_1_, diffraction data were collected to 2.2 Å resolution and the structure was solved by molecular replacement (Table 2). Each asymmetric unit contained a single copy of the 14-3-3-ζ/α4-30pS complex. Visible electron density for the α4 peptide was only present for 8 out of 30 residues (KRQYK**pS**IL) in the α4 peptide (Fig. 3a), suggesting a high degree of disorder for most of the α4 sequence. This KRQYK**pS**IL peptide corresponds to a mode-2 14-3-3 binding motif, except for the leucine in the pS + 2 position, which is a proline in the optimal motif sequence.^15^ The structure has similar features to those observed in previous 14-3-3-ζ/phospho-peptide complexes.^30^ The 14-3-3-ζ protein is a flat, W-shaped dimer, and each subunit contains nine antiparallel α-helices. The overall electrostatic surface of 14-3-3-ζ is negatively charged, but the channel accommodating the phospho-peptide has a basic pocket for the phosphate group (Fig. 3b). The α4 integrin peptide binds in this pocket, and the phosphate forms electrostatic interactions with the conserved K49, R56 and R127 residues with an additional hydrogen bond involving the Y128 side-chain hydroxyl (Fig. 3c). Other key 14-3-3 residues that participate in peptide binding are illustrated in Fig. 3d.

The 14-3-3-ζ/α4 complex is the second structure of an integrin tail bound to a 14-3-3 protein, after the previously described 14-3-3-ζ/β2-pT.^20^ As illustrated in Fig. 3e, the β2 integrin peptide conforms to a mode-1 14-3-3 binding motif with a phospho-threonine whereas the α4 integrin peptide is mode-2, featuring a phospho-serine; the arrangement of the two peptides is otherwise similar. PISA analysis^31^ gives an interface area of 559.2 Å^2^ for the 14-3-3-ζ/α4 complex and 449.6 Å^2^ for 14-3-3-ζ/β2, and an overlay of the two complexes shows that the two integrin peptides superimpose well, with most residues involved in complex formation having identical positions in the two structures (Fig. 3f).

### 14-3-3-ζ binding to the α4 integrin cytoplasmic tail and paxillin

The interactions of α4 and 14-3-3-ζ with paxillin were also investigated. The association of paxillin with α4 has been detected using pull-down experiments with glutathione *S*-transferase fusion proteins^22,32,33^ and, very recently, by NMR in a study that also reports the solution structure of the α4 integrin tail.^34^ Using a construct of the N-terminal region of paxillin (residues 1–323) that included all LD motifs (Supplementary Fig. 1a), we detected no specific interaction with the α4 integrin tail by NMR (Supplementary Fig. 1b) and addition of paxillin caused no further changes in a spectrum containing an α4/14-3-3-ζ mixture (data not shown). Similarly, no pairwise interaction was detected by ITC (Supplementary Fig. 1c and d). The possibility that a paxillin/14-3-3-ζ association depends on phosphorylation was also assessed. The ELM (*e*ukaryotic *l*inear *m*otif) server^35^ indicated three putative 14-3-3 binding motifs in the paxillin N-terminus; of these, only one has been reported to be phosphorylated *in vivo*^36^ (Supplementary Fig. 1a). A phosphorylated peptide containing this motif (corresponding to paxillin residues 111–128) was tested for binding to 14-3-3-ζ; however, no interaction was detected by ITC (Supplementary Fig. 1e). Thus, our biophysical studies do not support the direct involvement of the paxillin N-terminus in the association of 14-3-3-ζ and α4. The recent report of paxillin binding to α4 detected by NMR used shorter paxillin fragments than us, covering the LD2–LD4 region.^34^ It is possible that, in the whole N-terminal paxillin, the α4 interaction sites are masked, which would explain the absence of an interaction in our studies.

### Interaction studies of the β2 integrin tail with 14-3-3-ζ

**Fig. 4:**
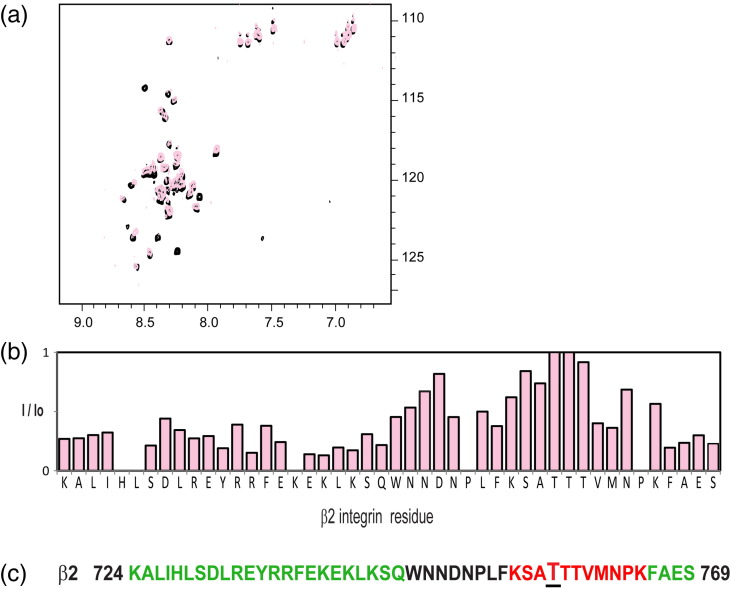
NMR titrations between β2 wt and 14-3-3-ζ. (a) ^15^N–^1^H HSQC spectrum of ^15^N-labeled 0.1 mM β2 wt in the absence (black) and presence of an equimolar amount of unlabeled 14-3-3-ζ (light pink). (b) The intensity of β2 integrin tail resonances observed in (a) plotted as the intensity ratio (*I*/*I*o) in the presence (*I*) and absence (*I*o) of 14-3-3-ζ. (c) Amino acid sequence of the β2 integrin cytoplasmic domain with the two 14-3-3-ζ binding sites for the phosphorylation-independent (green) and phosphorylation-dependent (red) sites denoted. The phosphorylatable T758 is shown in bigger font size and underlined.

To gain further insight into integrin tail binding to 14-3-3-ζ, we tested the interaction of the β2 integrin tail (residues 724–769) with 14-3-3-ζ in an analogous manner to the experiments with α4. NMR ^15^N heteronuclear single quantum coherence (HSQC) experiments of unphosphorylated β2 integrin tail in the absence or presence of 14-3-3-ζ were recorded. Addition of 14-3-3-ζ caused selective broadening of β2 resonances (Fig. 4a), revealing that the binding region is mainly located in the membrane proximal part of the integrin sequence and the last four C-terminal residues (Fig. 4b), with essentially no overlap with the previously described 14-3-3-ζ binding to β2 that occurs upon phosphorylation of T758 (Fig. 4c). This implies that the β2 integrin tail has two distinct 14-3-3-ζ sites within its sequence.

**Fig. 5:**
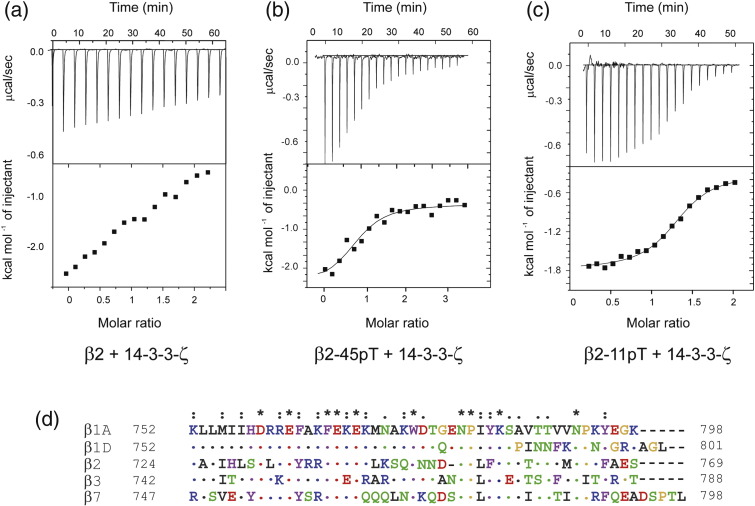
ITC of β2 phospho-peptide interactions with 14-3-3-ζ and sequence alignment of β-integrin tails. (a) ITC sensorgram of an in-cell 200 μM unphosphorylated β2 wt sample injected with 2 mM 14-3-3-ζ; the interaction is weak, in the millimolar range, and an accurate fit could not be obtained. (b) ITC sensorgram of an in-cell 120 μM β2-45pT phospho-peptide injected with 1.2 mM 14-3-3-ζ; thermodynamic parameters from the fit are shown in Fig. 2e. (c) ITC sensorgram of an in-cell 120 μM β2-11pT phospho-peptide injected with 1.2 mM 14-3-3-ζ. (d) Sequence alignment of β-integrin tails used in this study with residues colored depending on amino acid type (blue for basic, red for acid, purple for aromatic, black for aliphatic, green for polar and yellow for Gly and Pro residues). Asterisks, colons and dots indicate the degree of conservation. Residues identical with β1A in equivalent positions are indicated with dots.

The β2/14-3-3-ζ binding strength was also compared with that of α4/14-3-3-ζ. Similar to α4 wt, unphosphorylated β2 wt showed only a weak interaction by ITC (Fig. 5a). To evaluate the effect of phosphorylation on β2, we carried out ITC experiments on the interaction between 14-3-3-ζ and phosphorylated long and short β2 integrin peptides. *K*_d_ values of 8 μM and 4 μM were obtained for β2-45pT and β2-11pT, respectively (Figs. 2e and 5b and c). These results show that, unlike α4, a short 14-3-3-ζ binding motif in the β2 integrin tail is sufficient to give a relatively high affinity interaction and that contributions to the affinity from flanking residues outside the 14-3-3-ζ motif are much less significant in the β2/14-3-3-ζ than in the α4/14-3-3-ζ interaction. Thus, although the X-ray structures show a similar arrangement for the two integrin peptides in the 14-3-3-ζ complexes, there are significant differences in the binding characteristics of the two integrin tails.

### Interaction studies of 14-3-3-ζ and other β-integrin tails

Our observation that the membrane proximal region of the β2 integrin tail can interact in a phosphorylation-independent manner with 14-3-3-ζ raises the possibility that other β-integrin tails could behave similarly since sequence conservation is high across β-integrin tails in this region (Fig. 5d). In contrast, α-integrin tail sequences are much more divergent; for example, there is no serine or threonine equivalent to the phosphorylation site of α4 in other α-integrin tails. Thus, studies of other α-integrin tails were not pursued, but instead, we focused on β-integrin tails.

NMR ^15^N HSQC experiments with labeled β1A, β3 and β7 and unlabeled 14-3-3-ζ (Supplementary Fig. 2a–c) showed an intensity reduction for several resonances upon 14-3-3-ζ addition. Mapping the intensity changes reveals that binding occurs to β1A and β3, primarily in the N-terminal region, in a similar way to β2 (Supplementary Fig. 3a and b). The pattern of resonance intensity changes is more widespread in β7 (Supplementary Fig. 3c). Our results are consistent with a study that detected a phosphorylation-independent interaction between 14-3-3-β and β1A by yeast two-hybrid experiments.^21^ That study also showed that the β1A region containing serine/threonine residues was dispensable for the β1A/14-3-3-β interaction. Our NMR studies indicate that the β1D integrin tail, which has threonine-to-asparagine substitutions, interacts with 14-3-3-ζ in a very similar way to β1A (Supplementary Figs. 2d and 3d).

We also explored whether the phosphorylation-dependent 14-3-3-ζ binding site seen for β2 is present in other β-integrin tails. As illustrated in Fig. 3e, all β-integrin tails contain potential 14-3-3 binding motifs in their C-terminal regions. Unlike β2-11pT, no binding of peptide β1A-11pT was detected by ITC (data not shown) possibly because the mode-2 14-3-3 binding motif in β1A is too divergent from the canonical motif (apart from not having a proline in position pS/T + 2, the β1A motif also lacks an aromatic residue in position pS/pT-2, having instead an alanine) (Fig. 3e). It thus appears that the β2 integrin tail is unusual in having two distinct non-overlapping 14-3-3-ζ binding sites. β-Integrin tails with no phosphorylation site equivalent to that in β2 (such as β1A, β1D and β7) possibly only interact via the phosphorylation-independent binding mode.

**Fig. 6:**
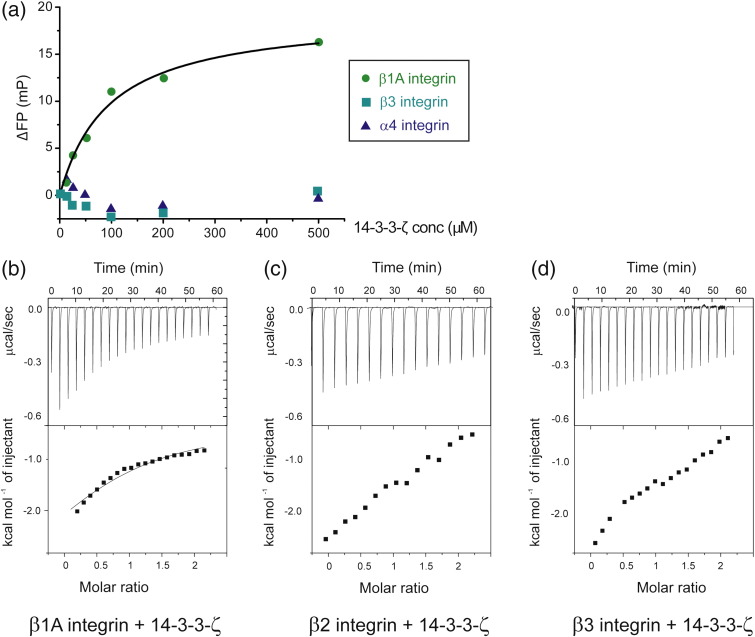
Fluorescence polarization and ITC of interactions between integrin tails and 14-3-3-ζ. (a) Fluorescence polarization titrations of fluorescein-labeled β1A, β3 and α4 with 14-3-3-ζ. No binding was detected for β3 and α4, but β1A clearly binds. The β1A titration was fitted to a single-site model as described elsewhere,^37^ with a value of *K*_d_ = 95 ± 16 μM. (b–d) ITC sensorgram of an in-cell 200 μM β1A, β2 (same as in Fig. 5a) or β3 samples injected with 2 mM 14-3-3-ζ. Only β1A gave a binding curve that could be fitted with *K*_d_ = 280 ± 40 μM.

Fluorescence polarization was used to compare the binding of different integrin tails to 14-3-3-ζ. Fluorescently labeled β1A, β3 and α4 integrin tails were titrated with increasing amounts of 14-3-3-ζ. As shown in Fig. 6a, a stronger interaction was observed between 14-3-3-ζ and β1A than with β3 or α4. These differences in affinity were later confirmed using ITC, where β1A shows a significantly stronger binding, with *K*_d_ ~ 280 μM, than β2, β3 and α4 (Figs. 2b and 6b–d).

### Specificity of 14-3-3-ζ/β1A integrin tail interaction

To gain further insight into the β1A/14-3-3-ζ association, we investigated the effects of 14-3-3-ζ substitutions on binding to β1A integrin tail. It has been reported that the substitution S58A in 14-3-3-β abolishes the β1A interaction.^38^ S58 regulates the dimeric state of 14-3-3,^39^ but it is not involved directly in binding to phospho-peptides. The binding of 14-3-3-ζ variants S58A and S58E to labeled β1A was studied using NMR. As shown in Supplementary Fig. 4, there is significant reduction in β1A resonance intensity change with the S58A variant compared to the wt; the intensities change even less with the S58E variant. A similar result was obtained with the β2 integrin tail (data not shown). These results confirm that S58 is an important residue for β-integrin tail interactions, either directly by participating in the binding or indirectly by abrogating the dimeric 14-3-3 state, thus explaining how phosphorylation of S58 could regulate these interactions.

Note that there were technical difficulties in making some of these measurements of weak affinity interactions. Some ITC measurements were limited by protein solubility. The NMR experiments, often one of the best ways of analyzing weak interactions, were limited because the exchange regime was often in the intermediate range, giving broad lines. The combined results of the ITC, NMR and fluorescence results were, however, all gave consistent relative values and results.

## Discussion

The interaction between the α4 integrin tail and 14-3-3-ζ has been characterized here using a range of biophysical techniques. The crystal structure of the phosphorylated α4/14-3-3-ζ complex shows that the α4 residues around the pS bind to 14-3-3-ζ in a mode similar to other 14-3-3-ζ/phospho-peptide complexes. Specific interactions for both unphosphorylated and a phospho-mimetic form of the α4 integrin tail with 14-3-3-ζ were detected by NMR, but ITC affinity measurements revealed that the 14-3-3-ζ interaction with unphosphorylated α4 integrin tail is much weaker than that with the phosphorylated forms. Residues outside the phosphorylated binding motif make important contributions to the interaction since binding of a short phosphorylated α4 integrin peptide is weak compared to a longer one.

The characteristics of the 14-3-3-ζ/α4 interaction reported here fit well with the hypothesis for 14-3-3/ligand complexes described by Yang *et al.* that involves primary and secondary interactions.^40^ Primary interactions involve contacts with residues around the phosphate group of the target protein associated with mode-1 and mode-2 binding motifs; secondary interactions are phosphorylation independent but crucial for ligand specificity.^41^ Our results suggest that significant secondary interactions occur in the 14-3-3-ζ/α4 complex. Only the primary interactions were observed in the X-ray structure with the secondary interactions presumably too dynamic to observe in the crystal.

Comparison of the published 14-3-3-ζ/β2 structure with our 14-3-3-ζ/α4 complex revealed similar peptide binding modes; however, the observation of phosphorylation-independent interactions for the α4 integrin tail and the importance of residues outside the 14-3-3 motif led us to further explore the 14-3-3-ζ/β2 interaction. A phosphorylation-independent interaction was found for β2 as well, and it was mapped to a location distinct from the phosphorylation-dependent 14-3-3-ζ binding region of that integrin tail.^20^ As in α4, this phosphorylation-independent interaction is much weaker than that mediated by the β2 phosphorylation site. However, the short phosphorylated binding motif in the β2 integrin tail has much higher affinity for 14-3-3-ζ than the corresponding α4 motif. While residues outside the 14-3-3 binding motif are important for an efficient interaction in α4, short and long β2 phospho-peptides associated to 14-3-3-ζ with virtually the same affinity. These different requirements for flanking residues could arise from cooperative effects for the α4 interaction, not present in the β2 case.

The NMR interaction studies were extended to β1A, β3, β7 and β1D; these indicated that 14-3-3-ζ binds all of these in a phosphorylation-independent manner. In β1, β2 and β3 integrin tails, the binding involves a stretch of 15–20 residues in the membrane proximal part. All these interactions were found to be very weak, but the β1A/14-3-3-ζ association was found to be significantly stronger than for other β-integrin tails.

Numerous regulated protein–protein interactions are essential in a functioning cell. Many of these interactions are weak and they are sensitive to secondary effects, for example, modulation of local concentration by sequestration of partners close to the plasma membrane.

Although the 14-3-3 interactions detected here are weak, many specific biologically relevant interactions of similar magnitude are known, for example, talin/β-integrin tail interactions.^3^ Presumably these affinities are tuned to be sensitive to modulation of local concentrations in the cell.^42,43^ Studies of these weak interactions are often contradictory because interactions detected by some methods, such as yeast two-hybrid and pull-down experiments with fusion proteins, are no longer detected when purified reagents and biophysical methods are applied.

It has been proposed that 14-3-3-β regulates integrin-mediated cell adhesion by a FAK-independent mechanism.^21^ Other studies reported that the absence of 14-3-3-ζ inhibited integrin-induced Rac1 activation and cell spreading and that these processes were compromised by a 14-3-3-ζ S58D variant.^44,45^ Our results with 14-3-3-ζ S58 variants are consistent with these findings and highlight the role of S58 in mediating 14-3-3-ζ interactions with integrins.

In conclusion, the crystal structure of an α-integrin cytoplasmic tail in complex with 14-3-3-ζ together with detailed biophysical studies have shown that, while many structural features are similar to other known 14-3-3 complexes, the binding exhibits subtle features involving secondary sites. We have also shown that 14-3-3-ζ is a general binder of β-integrin tails, with a preference for β1A, and that β2 contains two independent, non-overlapping 14-3-3-ζ binding sites. Questions beyond the scope of this work will be key for future studies: what is the physiological role of 14-3-3/integrin tail interactions; how do properties and affinities of the various 14-3-3/integrin tail complexes relate to different aspects of cell biology; the determination of the structure of a 14-3-3/unphosphorylated β-integrin tail complex would also provide valuable information about the molecular details of the novel β-integrin tail interaction we reported and whether this is likely to be involved in competition with talin in the process of integrin activation.

## Materials and Methods

### Protein expression and purification

Integrin cytoplasmic tails were cloned into a modified pET16b vector with an N-terminal His_10_ tag followed by a 3C protease cleavage site and the required integrin tail sequence and expressed in *Escherichia coli* BL21 (DE3) with 0.5 mM IPTG overnight at 37 °C. M9 minimal media containing ^15^NH_4_Cl and ^13^C glucose were used to prepare labeled integrin tails. The proteins were purified under denaturing conditions [50 mM sodium phosphate, 150 mM NaCl, 8 M urea and 0.035% β-mercaptoethanol (pH 7.0)] by Co^2 +^ affinity resin (Thermo Scientific) eluting with 200 mM imidazole. Samples were then dialyzed against 50 mM sodium phosphate, 150 mM NaCl, 1 mM ethylenediaminetetraacetic acid and 1 mM DTT, cleaved with 3C protease at 4 °C and further purified by reverse phase HPLC using a C_18_ column (Jupiter).

14-3-3-ζ was expressed from the same pET16b vector in an analogous manner (0.5 mM IPTG, 20 °C incubation). Cells were harvested by centrifugation; resuspended in 50 mM sodium phosphate, 150 mM NaCl and 0.035% β-mercaptoethanol (pH 7.0); and disrupted by sonication. The soluble fraction was purified by Co^2 +^ affinity chromatography, cleaved to remove the His_10_ tag and further purified by size-exclusion chromatography in NMR buffer [20 mM sodium phosphate and 50 mM NaCl (pH 6.5)], using a Superdex75 column (GE Healthcare).

Integrin tail and 14-3-3-ζ mutants were generated according to the QuikChange site-directed mutagenesis protocol (Stratagene) and produced and purified in the same way as wt proteins.

### NMR spectroscopy

Samples were prepared in NMR buffer (pH 6.5) with 5% D_2_O, and experiments were performed at 25 °C. Titration experiments were collected on a 500-MHz spectrometer using a water flip-back-embedded gradient enhanced ^15^N–^1^H HSQC pulse sequence.^46^ For the assignment of the α4 and β2 backbone resonances, ^15^N–^13^C integrin tails of 0.6 mM concentration were used to measure CBCA(CO)NH and CBCANH experiments on a Bruker 500-MHz instrument equipped with a cryogenic probe head. Data were processed using NMRPipe^47^ and analyzed with the programs CARA/XEASY^48^ or CCPN Analysis.^49^

### ITC and fluorescence polarization

Phosphorylated α4 synthetic peptides were purchased from GL Biochem Ltd. (Shanghai). All recombinant proteins and synthetic peptides were prepared in 20 mM sodium phosphate and 50 mM NaCl (pH 6.5), and concentrations were determined by UV absorbance at 280 nm. ITC experiments using a VP-ITC_200_ instrument (MicroCal) were performed at 298 K as follows: the cell (volume, ~ 200 μl) contained the different integrin tail fragments, and the syringe (volume, ~ 40 μl) contained 14-3-3-ζ. A first injection of 2 μl was followed by 15 injections of 2.5 μl, with a stirring speed of 1500 rpm and a delay between injections of 180 s. A blank titration was performed by injecting 14-3-3-ζ into buffer to take the heat of dilution into account. Experiments were repeated twice for each sample. Raw data were processed and fitted with MicroCal Origin software using a one-site model where the error function is calculated as the sum of the squared deviations between the data and the model curve.

For fluorescence polarization experiments, integrin tails were labeled with fluorescein-5-maleimide (Invitrogen) linked to an engineered C-terminal Cys residue. Measurements were performed using 96-well microplates (Corning) in a PHERAstar FS microplate reader (BMG Labtech). Fluorescein-labeled integrin tails at 1 μM concentration were excited at 485 nm, and polarization was recorded at 520 nm.

### Crystallization, data collection and structure determination

Samples for crystallization trials contained 10 mg/ml (0.35 mM) 14-3-3-ζ and 2.6 mg/ml (0.7 mM) α4-30pS peptide in 20 mM Tris–HCl and 50 mM NaCl (pH 7.5). Crystals were grown by the sitting-drop vapor diffusion method at 4 °C using 1:1 ratios of sample and 0.1 M Na Hepes (pH 7.5) and 25% polyethylene glycol 2000 monomethyl ether mother liquor. For data collection, crystals were soaked in the same buffer plus 15% glycerol and flash frozen in liquid nitrogen.

Data were collected at the Diamond Light Source beamline I04-1; the diffraction data extended to 2.2 Å resolution. Data were indexed and integrated using MOSFLM and merged using SCALA from the CCP4 program suite.^50^ A subset of approximately 5% of total reflections were flagged for use in calculating *R*_free_. Initial structure determination was performed by molecular replacement using Phaser^51^ with Protein Data Bank entry 2O02^52^ as the search model. Initial refinement was performed with PHENIX,^28^ model building was performed with Coot^53^ and subsequent refinement was performed with BUSTER.^54^ The structure refined to a satisfactory *R*_work_/*R*_free_, and the quality of the structure was assessed using MolProbity.^55^ Omit maps were calculated using PHENIX.^28^ Table 2 provides the crystallographic data and refinement statistics. PyMOL^56^ and the PISA server from European Bioinformatics Institute^31^ were used to analyze the structure and prepare the structural figures.

### Accession codes

Atomic coordinates for the 14-3-3-ζ/α4 phosphorylated peptide complex have been deposited in the Protein Data Bank under accession number 4HKC, and chemical shift resonance assignments for the α4 and β2 integrin cytoplasmic domains have been deposited in the Biological Magnetic Resonance Bank under accession numbers 18718 and 187191871818719.

The following are the supplementary materials related to this article.

1433_a4_complex

**Figure:**
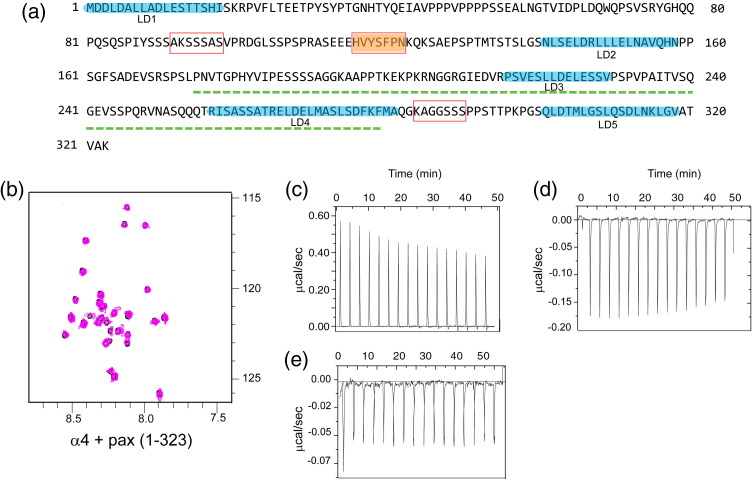
Supplementary Fig. 1.

**Figure:**
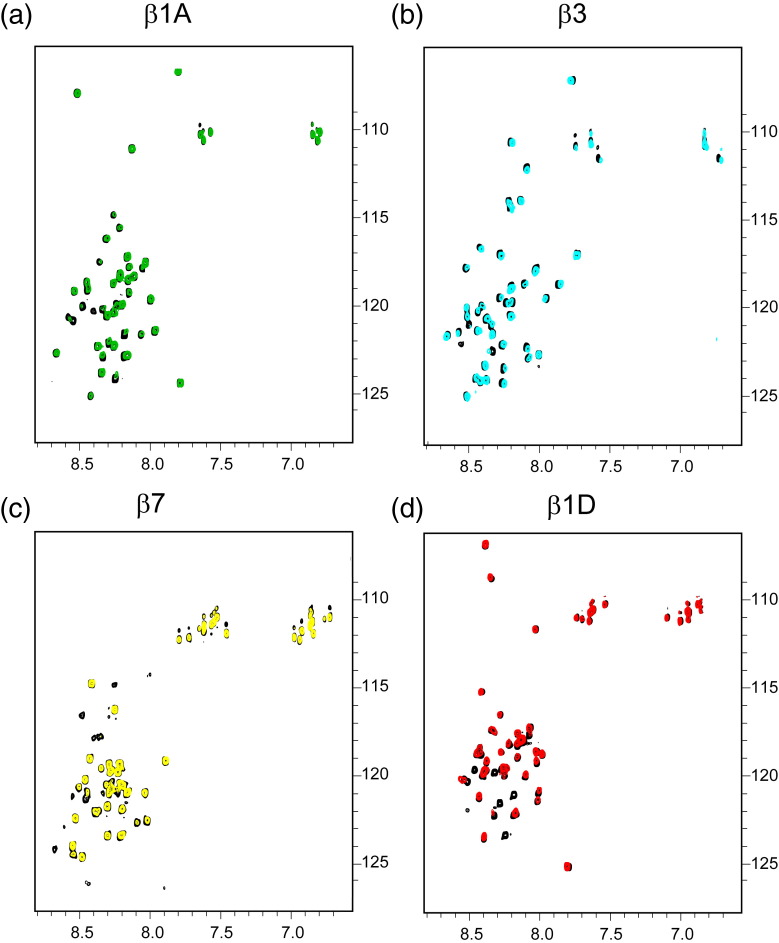
Supplementary Fig. 2.

**Figure:**
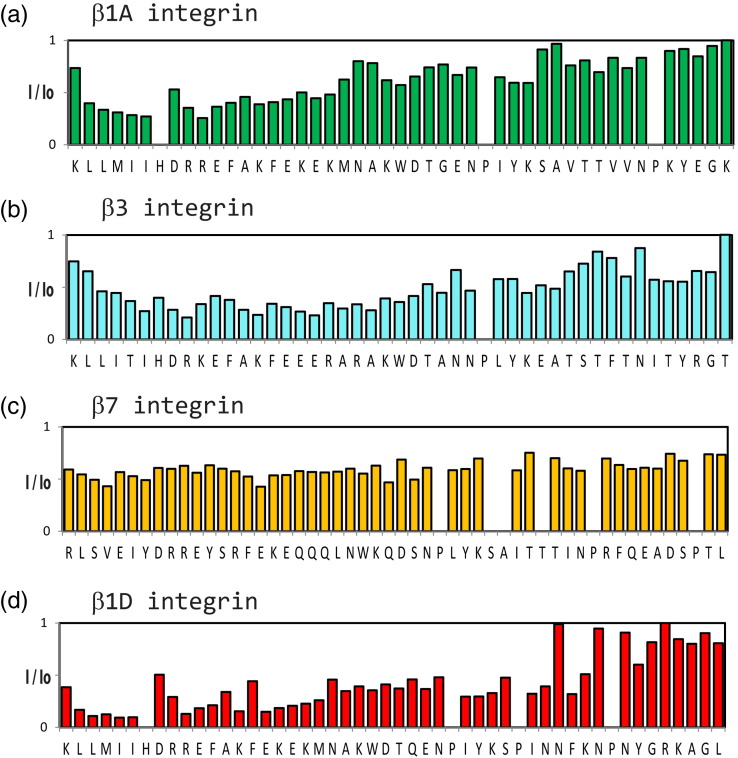
Supplementary Fig. 3.

**Figure:**
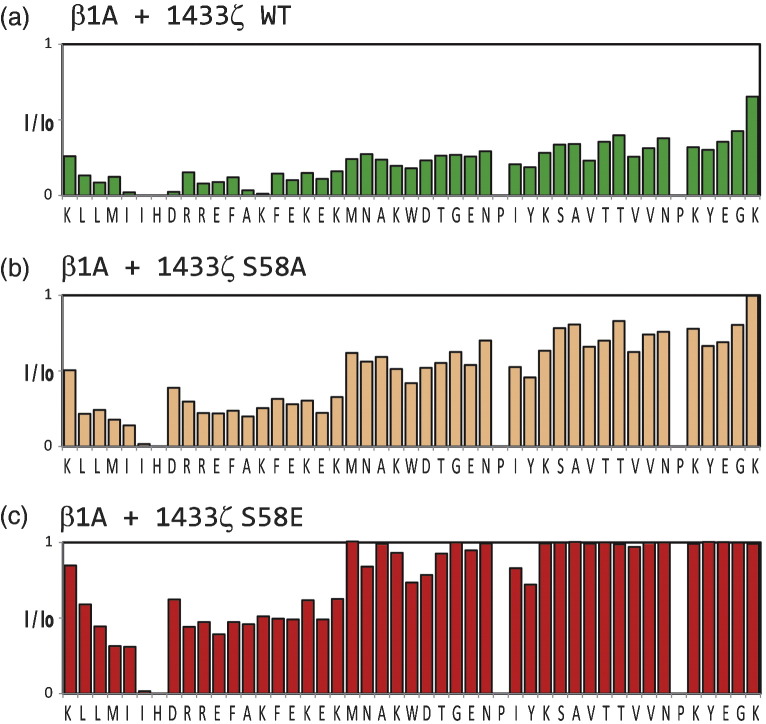
Supplementary Fig. 4.

## Figures and Tables

**Table 1:** Integrin constructs used in experiments

Name	Residue numbering^a^	Sequence^b^	Source^c^
α4 wt	1001–1032	*GPLGS*KAGFFKRQYKSILQEENRRDSWSYINSKSNDD	R
α4 S1011D	1001–1032	*GPLGS*KAGFFKRQYK**D**ILQEENRRDSWSYINSKSNDD	R
α4-30pS	1003–1032	GFFKRQYK**pS**ILQEENRRDSWSYINSKSNDD	S
α4-11pS	1005–1015	FKRQYK**pS**ILQE	S
β2 wt	724–769	*GS*KALIHLSDLREYRRFEKEKLKSQWNNDNPLFKSATTTVMNPKFAES	R
β2-45pT	724–769	KALIHLSDLREYRRFEKEKLKSQWNNDNPLFKSA**pT**TTVMNPKFAES	S
β2-11pT	752–762	PLFKSA**pT**TTVM	S
β1A wt	752–798	*GS* KLLMIIHDRREFAKFEKEKMNAKWDTGENPIYKSAVTTVVNPKYEGK	R
β1-11pT	783–793	YKSAV**pT**TVVNP	S
β1D wt	752–801	*GS* KLLMIIHDRREFAKFEKEKMNAKWDTQENPIYKSPINNFKNPNYGRKAGL	R
β3 wt	742–788	*GS* KLLITIHDRKEFAKFEEERARAKWDTANNPLYKEATSTFTNITYRGT	R
β7 wt	747–798	*GS* RLSVEIYDRREYSRFEKEQQQLNWKQDSNPLYKSAITTTINPRFQEADSPTL	R

a According to Uniprot entries P13612, P05107, P05556, P05106 and P26010P05106P26010.

b pS and pT correspond to phospho-serine and phospho-threonine, respectively, and are in boldface together with serine/threonine substitutions to aspartic. Amino acids from the cloning tag in the N-terminus of the sequence are italicized.

c R, recombinant; S, synthetic.

**Table 2:** Crystallographic data collection and refinement statistics

*Data collection*	
Beamline	Diamond, I04-1
Wavelength (Å)	0.9163
Space group	*C*222_1_
Cell parameters	
*a*, *b*, *c* (Å)	89.03, 111.60, 72.99
α, β, γ (°)	90, 90, 90
Resolution (Å)	44.50–2.20 (2.32–2.20)
Total reflections	110,654 (15,795)
Unique reflections	18,798 (2725)
*R*_merge_	0.079 (0.394)
Completeness (%)	99.8 (100)
Multiplicity	5.9 (5.8)
*I*/σ(*I*)	9.8 (3.2)

*Refinement*	
*R*_work_/*R*_free_ (%)	19.7/23.9
RMSD from ideal values	
Bonds/angles (Å/°)	0.010/1.02
Overall mean *B* values (Å^2^)	
Protein	59.2
Peptide	70.3
Solvent	60.2
No. of amino acid residues per asymmetric unit	237
No. of water molecules	121
Matthews coefficient	2.79 (solvent content, 55.93%)
MolProbity statistics	
All-atom contacts: clashcore, all atoms	1.58
Protein geometry: poor rotamers (%)	0.50
Ramachandran plot (%)	
Residues in preferred regions	98.3
Residues in allowed regions	1.7
Residues in disallowed regions	0.0
C^β^ deviations greater than 0.25 Å	0
MolProbity score	0.90
Residues with bad bonds (%)	0.0
Residues with bad angles (%)	0.0

Values for the highest-resolution shell are shown in parentheses.
